# Influence of Overlap on Surface Quality in the Laser Polishing of 3D Printed Inconel 718 under the Effect of Air and Argon

**DOI:** 10.3390/ma14061479

**Published:** 2021-03-17

**Authors:** Michał Ćwikła, Robert Dziedzic, Jacek Reiner

**Affiliations:** Faculty of Mechanical Engineering, Wroclaw University of Science and Technology, Wybrzeże Stanisława Wyspiańskiego 27, 50-370 Wroclaw, Poland; robert.dziedzic@pwr.edu.pl (R.D.); jacek.reiner@pwr.edu.pl (J.R.)

**Keywords:** Laser Polishing, Additive Manufacturing, oxidation layer, statistical analysis, Inconel 718

## Abstract

Laser Polishing (LP) is a well-defined technology that has recently been applied to improve three-dimensional (3D) printed Inconel 718 (IN718) parts. However, the necessity to conduct the process in an argon chamber is one of its major drawbacks, which is associated with an increase in the costs of production and the limitations of the technology regarding the size of parts that can be polished. This article investigates the possibility to conduct LP of IN718 in an air atmosphere and compares the results with those from an argon chamber setup. The experiment was carried out in the context of the influence of overlap on the final surface. The improvement of surface quality was defined through the evaluation of average areal roughness parameters, material relocation, periodic surface components, and the categorization of process-induced structures. It was found that LP allows for the average roughness to be reduced by 82.8% and 87.9% for an air and argon atmosphere, respectively. The oxidation layer was characterized using Energy-Dispersive X-ray Spectroscopy (EDS) analysis. The formation of overlap with regards to Ti and Al oxides had a vital influence on surface quality.

## 1. Introduction

Additive Manufacturing (AM) technologies are extensively developed in the fields of aerospace, medicine, and industry [1]. Selective Laser Melting (SLM) is a commonly used type of AM. Parts are formed during layer-by-layer re-melting of the metal powder using a laser beam and galvo-scanner [2]. Currently, SLM as-built parts, after process optimization, are characterized by a homogenous structure, low porosity, and a lack of discontinuities, cracks [3], and pores [4]. However, the surface of an as-built part after the AM process is still characterized by a roughness that is not acceptable for most applications (ranging from 5 to 15 µm). This roughness not only depends on the process parameters, but also on the direction of printing [5]. Therefore, there is often a need to apply polishing methods to improve the surface quality after fabrication. Apart from conventional hand-finishing, electrochemical [6] and ultrasonic abrasive [7] methods have been successfully adopted so far to reduce the roughness of three-dimensional (3D) printed parts.

One technology, which has been recently extensively developed in the context of polishing 3D printed, is Laser Polishing. It is a contact-less method that provides high speed, high precision, and full automation machining. The idea is to locally scan the surface with a laser beam with a laser intensity high enough to cause the formation of a melt-pool. Surface tension, viscosity, and gravity lead to the redistribution of the material and, therefore, the peaks and valleys are flattened, and the roughness is reduced. The material is generally not removed, and is instead relocated, as opposed to other polishing methods [8]. Depending on the initial surface topography and laser energy density, two main polishing mechanisms can be distinguished. In Shallow Surface Melting (SSM), the re-melting zone is shallower than the peak-to-valley depth. This is due to the fact that, under the action of the capillary pressure and the liquid curvature, the molten material fills the “valleys” in between. The transition to Surface Over Melt (SOM) occurs at some point with an increasing power density. The melting zone is extended above the height of the surface asperities, and an increasing temperature causes the surface tension gradient to overcome the viscous forces. As a result, a low frequency ensues, and periodic structure is formed [9,10].

In the context of LP, pulsed lasers working at a nanosecond regime are usually utilized in order to improve the surface quality of the surface that is already characterized by a low initial roughness <1 µm (e.g., after micro milling and grinding). When pulsed nanosecond lasers are used, re-melting depths are usually in the range of 0.5–5 µm, and material is redistributed and solidified before the next pulse hits the surface. Such a process is named Laser Micro Polishing (LµP), where the SSM mechanism usually occurs. However, CW lasers are used to create a constant, moving liquid pool on the surface. Their re-melting depths are in the range of 20 to 200 µm, and they are therefore used for the LP of a high initial surface roughness (>1 µm). Such a process is called Laser Macro Polishing or Continuous Wave Laser Polishing (CW-LP), which is usually related to the SOM mechanism. For most metal CW-LP applications, Nd:YAG and fiber lasers are used due to their range of available power, initial and exploitation costs, and versatility. They generate a beam that is characterized by a satisfactory absorption for most metals [9,11,12].

**Figure 1:**
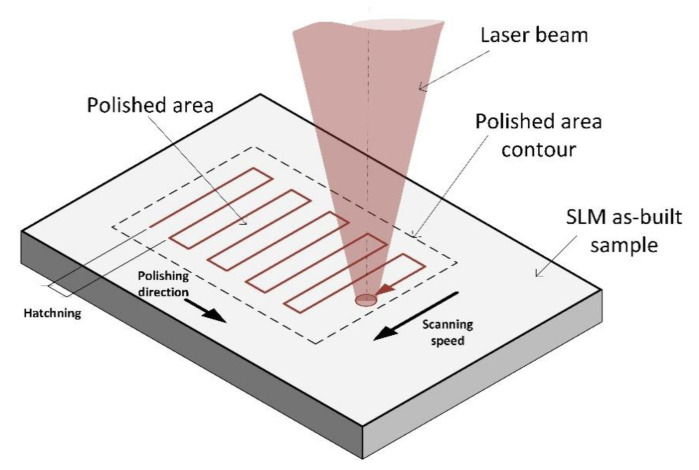
Zigzag strategy in context of Laser Polishing.

The most common scanning strategy for LP is the zigzagging of parallel lines (Figure 1) [13]. However, this has many drawbacks, such as the overall high heat impact (due to the required overlap between tracks), and the formation of step structures, ripples, bulges, and undercuts [14,15]. The distance between neighboring tracks is kept constant and it is called the hatching pitch (*h*). When its value is less than the diameter of the laser beam (*D*), the overlap (*OL*) can be calculated while using Equation (1) [12].

(1) $$OL=\left(1-\frac{h}{D}\right)\times 100$$

Because the energy density of the laser beam is not uniformly distributed on the surface, the overlap is needed in the case of LP based on re-melting [12]. However, the zigzag strategy is related to heat accumulation in the turning points, in turn causing the curb effect [16]. Thus, the topography from the boundaries can differ from the inner surface. A partial solution for this issue can be power ramping, or the monitoring and controlling of the laser power in a closed-loop. For the purpose of this experimental study, the edges of the polished area were not measured and considered. Recently, new approaches of designing beam trajectories have been presented to overcome some of the limitations of the zigzag strategy. Zhou et al. proposed the dual-beam LP of 3D complex surfaces. The authors used CW laser propagating in a zigzag trajectory and added a pulsed laser beam that propagated simultaneously in the square trajectory. The solution allowed for the efficiency of LµP to be significantly increased [17]. The recent works of Caggiano et al. [18] and Vittorio et al. [19] showed that adopting the trochoid trajectory from laser welding to LP gives promising results in context of roughness reduction. However, in these works, the results were not compared to the zigzag strategy. Vadali et al. proposed intelligent scanning trajectories that were driven by the artificial potential fields method. The authors’ approach allowed for the scanning trajectory to be adopted to the initial surface quality, therefore avoiding the residual, periodic patterns that are typical for the zigzag strategy [15]. Although different trajectory approaches seem to be promising, the topic is still only briefly covered and the utilization of some of these methods could be the subject of different research. Thus, in this paper, the well-known and investigated zigzag strategy was used to implement LP on the samples.

In terms of CW-LP and zigzag strategy, the term “overlap” concerns the distance between parallel laser lines, while, in LµP, where pulsed lasers are used, the overlap value is also calculated along the scanning direction and, therefore, depends on the scanning speed, beam diameter, pulse rate frequency, and pulse duration [20,21]. Rosa et al. showed that overlap is a vital parameter in the case of the optimization of the CW-LP process [22]. Bhaduri et al. investigated the LP of 3D printed parts that were made of 316L steel [21]. The authors achieved the greatest surface roughness reduction for 95% overlap (along the scanning direction). Hafiz et al. vastly investigated the influence of overlap on surface quality. The authors found that the surface quality improves up to 95% of the overlap for H13 tool steel. The authors noticed that the optimization of the overlap value should be made for a particular combination of other LP parameters [23]. Rosa et al. reported that the overlap parameter has an impact on the surface integrity and surface roughness. Moreover, the authors highlighted that increasing overlap from 60% tends to create micro-cracks in the case of the CW-LP of 3D printed 316L steel parts [24]. Yung et al. showed that the hatching distance can not only have an influence on roughness, but also on the wettability of the surface after LP [25].

It is generally said that an achievable surface topography and morphology depends strongly on three factors: (1) the thermal and optical properties of the material and the initial surface quality of the workpiece, (2) the laser radiation and optic parameters (such as laser power, beam diameter, and intensity profile), and (3) motion related parameters (such as scanning trajectory, velocity, and overlap percentage). Points 2 and 3 cover the process parameters that are controllable by the operator. In most papers concerning CW-LP, Energy Density (*ED_single_*, units: J × mm^−2^) is the parameter that is considered to be vital and the most influential on the final results. It is described as the ratio of the laser power (*P*, unit: W) to the beam’s diameter (*D*, unit: mm) and laser beam’s scanning speed/feed rate (*V_scan_*, unit: mm × s^−1^) (Equation (2)) [12,26]. This equation is true for Laser Polishing that is conducted on the single laser track. However, when multiple tracks overlap in zigzag strategy (*h* < *D*), Energy Density (*ED*, units: J × mm^−2^)) equation is modified: *D* in Equation (2) is replaced by *h* and the equation is multiplied by overlay ratio *x* = *D*/*h* (Equation (3)) [27].

(2) $$ED_{single}=\frac{P}{D\times V_{scan}}$$

(3) $$ED=\frac{P}{h\times V_{scan}}\times \frac{D}{h}$$

In the context of LP, the oxidation of the surface is generally considered to be unwanted, and, therefore, it was reported that shielding gas plays a major role in both CW-LP and LµP. Bharduri et al. conducted LµP of 316L in an air and argon environment, and reported that a similar roughness reduction can be achieved in both cases. Moreover, the authors pointed out that the color of the surface is directly related to the surface oxidation level. Conducting LP in a shielding environment may result in a gloss effect for low energy densities, while, in air, the surface appears gray/black. However, it was shown that laser-induced oxidation may be a desirable effect in some applications [21]. Temmler et al. noted that the utilizing of CO_2_ for the LP of AISI H11 tool steel may reduce the formation of martensite, and also that a higher roughness reduction can be achieved when compared to an Ar atmosphere [28]. Temmler et al. presented an extensive comparison of the influence of shielding gas on LµP. In the study, the LµP of 410 stainless steel was conducted with nitrogen, argon, and air atmospheres. The authors stated that nitrogen and argon provide similar results in terms of surface quality improvement, whereas the utilization of no shielding gas led to a higher micro-roughness [29]. Perry et al. reported microcracking of the surface when Laser Polishing Ti6Al4V in ambient air. They also stated that argon can be used to reduce the effect of micro-cracking [30]. Solheid et al. conducted LP without shielding gas and stated that the oxidation of laser polished 18Ni Maraging steel has an influence on its final topography [31]. Bures and Zetek conducted CW-LP on 3D printed IN718 and observed the formation of oxides. They also noted that the oxidation layer effects the surface roughness [32]. Therefore, most of the LP experiments are conducted in an argon atmosphere to prevent oxidation of the surface. However, the literature review shows that, in specific cases, it is possible to apply a different gas, or even no gas, and still achieve satisfying results. In the context of the LP of IN718, no studies have been carried out that compare the influence of shielding gases on the polishing results.

Surface topography analysis in many works proves that LP leads to roughness reduction with regards to high frequency components. However, when CW-LP is applied, redistributed material may form new, low frequency periodic components (waviness). This phenomenon is related to the SOM mechanism. [12,23]. Moreover, when the zigzag scanning method is applied, the surface is characterized by a periodic structure perpendicular to the scanning direction. This effect was noticed by many researchers and categorized by Nüsser et al. as bulges [14]. The wavelength of the structure is equal to the hatching pitch. However, when the overlap is increased significantly (above 80%), the periodic bulges are no longer dominant and, as a result, the higher frequency components are clearly amplified. Therefore, the roughness measured perpendicular to the scanning direction may be increased. In conclusion, LP induces new periodic surface components of high and low frequency, the identification of which is vital due to the fact that they contribute to both the roughness and waviness of the polished sample. Perry et al. proposed the application of statistical analysis in the spatial frequency domain, which allows periodic features of the surface, by conducting Fast Fourier Transform (FFT) on the extracted profile, to be detected [33]. This allowed for the periodic character of a single profile, or averaged values of perpendicular profiles, to be detected [23]. Willenborg [34] proposed a method of calculating the Sa in the frequency domain, with the method being further utilized in several LP works [29,35,36]. In this method (that is also known as the Sa-spectrum), the arithmetical mean height of the surface values (Sa) is calculated on data sets divided by phase-correct band pass filters into discrete spectral ranges. Thus, it allows for surface roughness features in the micro, meso, and macro regime to be determined. Hafiz et al. pointed out that the surface after LP is not only characterized by different periodical components, but also by a different distribution of height asperities [23]. Thus, the authors point out that the Abbott–Firestone curve (also known as the BAC and Material Ratio curve) enables the percentage of the material located below and above the reference line to be excluded. Krishnan et al. also highlighted the importance of this analysis [12].

Laser Polishing is successfully applied to most common materials in powder-based AM [20]. Inconel 718 (IN718) is one of the major materials to be polished, which, due to its high corrosion resistance and high strength at elevated temperatures (up to 650 °C), is widely used in high-temperature and high-pressure applications in the aerospace industry [35,36]. In some jet engines, IN718 is used for more than 50% of the parts, i.e., as the stator vane, gas turbine, diffuser case, and drum rotor [37]. However, conventional machining of IN718 is associated with significant tool wear, low material removal rates, and high material wastes [38]. AM technologies are overcoming these limitations, but they still provide a poor quality of the surface [39], which may lead to significant air flow disturbance in the case of jet engine stator vane blades. Thus, there is often a necessity to locally polish the surface after the fabrication process [40].

Recent Laser Polishing studies have shown that it is possible to achieve a reduction of roughness from 80% to 98% for additive manufactured IN718 samples [38,40,41,42,43,44,45]. However, in all of the mentioned papers, the LP of IN718 is conducted in an oxygen-free environment due to the fact that IN718 contains elements characterized by a high oxidation potential (Ni, Fe, Cr, Ti, Al) [39], which can form oxides in high temperatures both below [46] and above the melting point (1336 °C) [47]. In most cases, to provide an oxygen-free environment during LP, a SLM chamber filled with argon is used in order to proceed with LP directly after 3D printing [44]. However, in this case, the problem of object/laser beam manipulation limits, or even prevents, the polishing of complex parts and, therefore, the polishing can only be applied to flat horizontal surfaces. In other embodiments, a stand-alone station (such as Alpine from Fraunhofer ILT [48]) is used for the LP process and it allows for the sample and beam to be manipulated in six or more axis, which allows LP to be applied for complex parts [16]. However, this solution is related to the high initial costs, with the space in the chamber being limited to only the LP of small or medium parts. In many LP studies, a galvo-scanner is used and the laboratory set-up consists of a separated argon chamber [21,23,40,43]. This solution allows for the chamber or laser head, depending on the desired application, to be independently changed. However, the use of a chamber still limits the size of the processed parts to small and medium. Temmler et al. [29] also proposed a solution to coaxially deliver shielding gas though a nozzle that is regularly used in laser applications (e.g., cutting, welding). This allowed the limitation that is related to the use of a chamber to be overcome, but it prevented the use of the galvo-scanning system, therefore decreasing the efficiency and versatility of the process. Moreover, this solution can be mainly applied to simple surfaces that are preferably flat or cylindrical.

Krishnan et al., in their LP review, point out that high production costs and the oxidation of metal surfaces are some of the key challenges concerning the development of current LP methods [12]. LP in an oxygen-free environment prevents the surface from forming unwanted oxides; however, this results in higher production costs that are related to chamber usage and shielding gas wear. Moreover, some SLM parts can reach huge dimensions, and it can be difficult and expensive to provide a suitable chamber solution. Thus, the ability to conduct LP without a chamber could be a better method from the perspective of industry. 

This paper presents a comparison of IN718 surface topographies and morphologies after LP. The experiments were conducted with different overlap values in a shielding and air environment, therefore investigating the possibility to overcome limitations related to a chamber filled with shielding gas (argon). The characterization of the surface includes chemical analysis of the oxidation layer and topography analysis, such as: the evaluation of roughness, periodic components, and material relocation. 

## 2. Materials and Methods

### 2.1. Characterization of SLM Samples 

3D printed samples, which were later subjected to the LP experiment, were fabricated using a SLM 280 2.0 machine (SLM Solutions Group AG, Lübeck, Germany) that operates with a 1070 nm fiber laser up to 700 W, and that has a changeable focus diameter from 80 to 115 μm. Inconel 718 powder (SLM Solutions Group AG, Lübeck, Germany), characterized by a fraction size of 15–45 μm, was used to manufacture 100 mm × 100 mm × 3 mm flat bars. Printing was conducted on a steel platform (100 mm × 100 mm × 20 mm) that was preheated to 200 °C in order to avoid deformation of the fabricated part. During the process, the SLM chamber was filled with argon gas to provide a protective atmosphere, thus preventing oxidation. Table 1 presents the exact process parameters. After printing, the samples were annealed for 6 h in the temperature of 1150 °C in order to reduce residual stress.

### 2.2. Laser Polishing Station and Beam Caustic

**Figure 2:**
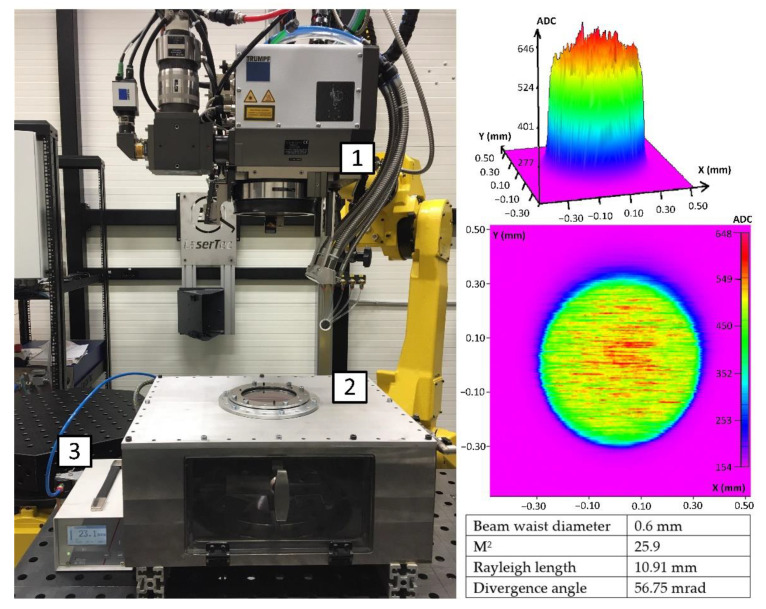
Experimental set-up utilized for the LP, 1-scanning laser head, 2-argon chamber, 3-oxygen analyzer (**left**). Beam intensity distribution in the focal plane and beam parameters (**right**).

In this paper, the fabricated samples were characterized by an initial roughness >1 µm (measured in Section 3.2 and a height of surface asperities much greater than the maximum re-melting depths that are available for LµP. Thus, a continuous wave laser was utilized to conduct the LP. The TruDisk 8001 (Trumpf, Ditzingen, Germany) solid-state Yb:YAG laser, emitting radiation up to 8 kW with a central wavelength of 1030 nm, was connected via optical fiber to a dedicated laser galvo head: PFO-33 (Trumpf), mounted on a six-axis robotic arm M-710iC70 (Fanuc) (Figure 2 left). The PFO-33 was positioned at the distance of 450 mm from the sample, which is equal to the effective focal length of the f-theta lens. Additionally, the Crossjet TL120 S0.25 (Trumpf) was used to protect the objective from the spatters and vapor from the process. The air pressure blowing though the crossjet nozzle was 4.5 bar, and the distance from the nozzle to the objective was set to 140 mm. The beam parameters were calculated from the laser beam caustic that was measured using the FocusMonitor (Primes, Pfungstadt, Germany) system. The beam’s waist and parameters were compiled using LaserDiagnosticsSoftware (Primes, version 2.98.81), with the results being presented in Figure 2 (right). The system uses a laser beam that was characterized by a top-hat distribution, which is generally considered to be more suitable for LP applications due to the fact that there is a more uniform temperature distribution on the surface [9].

### 2.3. Polishing Strategy and Overlap

Based on the literature review, it can be stated that the choice of the best overlap value strongly depends on the process conditions, i.e., laser parameters and regime (CW/pulsed), material properties, and surface quality. Thus, overlap should be determined for every different LP experiment. In this paper, seven different overlap values in the range of 50% to 90% were examined (Table 2) for an argon (a) and oxygen (o) atmosphere. There was no beam defocus and, therefore, the beam diameter was 0.6 mm, as measured in Section 2.2. The experiment was conducted with constant values of both laser power and scanning speed. These parameters were determined in the author’s preliminary experiment that considered a single re-melting line. For CW-LP, high heat input usually occurs, which is considered to be unwanted and may cause the surface to deform. Therefore, the laser power and scanning speed were defined in order to achieve the complete re-melting of asperities, and not to overheat the surface. 

### 2.4. Process Gas

A chamber (Figure 2) with dimensions of 500 mm × 400 mm × 200 mm was used to provide the argon atmosphere. The laser beam could enter the inside through the inspection window, which is characterized by a high transmission for the laser beam’s wavelength. Further LP experiments were conducted when the oxygen content stabilized to 25 ppm (±2 ppm), which was confirmed by readings from the BA-4510 oxygen analyzer (Beuhler Technologies, Ratingen, Germany). For the experiment without the shielding atmosphere, the process was conducted in an ambient air atmosphere. Additionally, the polished area was blown out with air flowing from the MDE-nozzle (Trumpf) that was mounted to the PFO-33 laser head. This procedure is commonly used in laser conduction welding and it allows for the metal vapor effect to be reduced and melt-pool formation to be stabilized. 

### 2.5. Surface Analysis

In this study, the topography measurements were performed using the Talysurf CCI (Taylor Hobson, Leicester, United Kingdom) white-light interference microscope that was equipped with a 20× CF IC objective (Nikon, Tokyo, Japan). The system is characterized by an optical resolution of 0.01 nm (z axis) and spatial resolution of 0.415 µm (x&y axis). Each measurement was preceded by cleaning the sample using IPA, and then purging with compressed air. Subsequently, a three-dimensional data set was analyzed using TalySurf software (Taylor Hobson, version 7 Gold).

Sa and Sq parameters were used for a basic comparison of roughness reduction. The procedures of determining these amplitude areal parameters were carried out in accordance with ISO 25178. Thus, the roughness parameters were calculated on the S-L surface extracted from the primary surface by applying a Gaussian filter (L-filter) with a wavelength equal to the nesting index (0.8 mm for polished surfaces and 2.5 mm for as-built surfaces). For the initial surface of the IN718, the Sa was equal to 5.86 µm and the Sq was equal to 8.07 µm. The Abbott–Firestone curve (ISO 25178) was then used to reveal the areal information about the relocation of the material, thus determining the predominance of the valleys or peaks in the polished surface. For every sample, the curve was evaluated for a 4 mm × 4 mm area. Periodic components of the surface, which arose after applying the LP, were determined using the Power Spectrum Density function. This function is the square of the FFT’s amplitudes, and it is represented by the wavelength domain for better understanding and clarity. For the polished samples, the presented PSD curves were the average of the PSD curves that were calculated along every horizonal cross-section (perpendicular to the scanning direction) on a 4 mm × 4 mm area. The number of cross-sections was over five thousand, which was related to the measured area size and the spatial resolution of the microscope.

Later, the EVO-25 SEM system (Zeiss) was used to enrich the visual inspection and to reveal more details about the surface morphology. The system was integrated with EDS (Brucker, Billerica, MA, USA), which allowed for the chemical characterization of the sample to be specified, therefore determining the chemical composition and oxidation level of the formed oxidation layer.

## 3. Results

**Figure 3:**
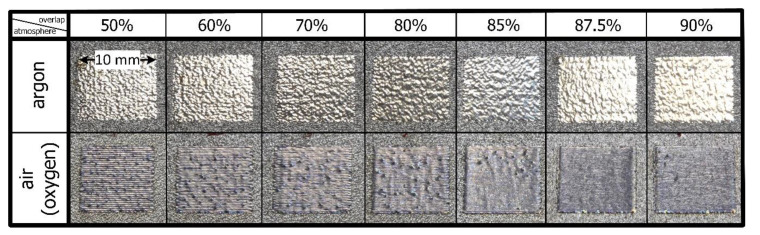
Physical appearance of the laser polished samples, proceeding in accordance with Table 2.

A preliminary visual inspection (Figure 3) revealed that the gloss effect was achieved for all of the samples that were polished in the argon chamber. However, it can clearly be seen that, although the surface is smooth, it is not completely flat. On the other hand, the samples that were polished in the air atmosphere appear to be dark due to the formation of an oxidation layer. Moreover, the gloss effect is no longer dominant.

### 3.1. Oxidation Layer

**Figure 4:**
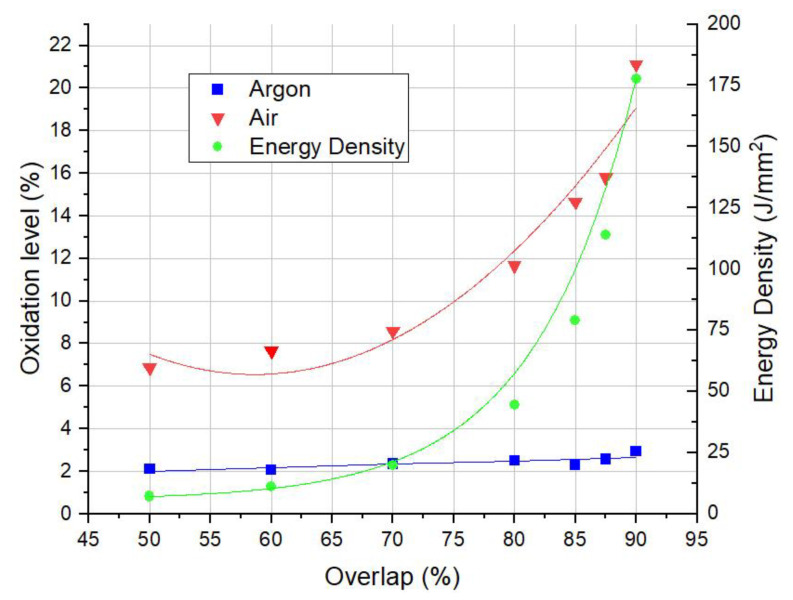
Surface oxidation level of the samples produced in accordance with Table 2 and the Laser Energy Densities values used in the experiment.

The EDS analysis was conducted on a 4 mm × 4 mm area on every sample from Table 2. Areal analysis allowed for the oxygen content on the polished surface to be estimated and, therefore, the oxidation level of the surface to be determined (Figure 4). Generally, EDS is not a perfect solution of performing quantify estimation of light elements (such as oxygen or carbon) with high accuracy. Instead, these analyses were only made within the context of qualitative studies and, thus, to compare the oxidation level for different samples. It is worth mentioning that, due to the EDS principle of operation, the chemical analysis in this section is not performed on the whole oxidation layer, but, instead, on a thin layer of approximately 1 µm. When the process is conducted in the argon chamber, the overlap has a minor influence on the oxidation level, slightly varying from the oxidation level on the initial surface (measured for approximately 2%), as shown in Figure 4. However, when no shielding atmosphere is applied, oxidation increases exponentially with the overlap. An explanation for this would be that the change in overlap is related to the ED and processing/interaction times. This simply means that for the same area of 1 cm^2^, and the same laser parameters (power, scanning speed), more energy is applied, in turn causing higher local heat accumulation on the surface to occur [28]. Moreover, with regards to the oxidation kinetics, the mass gain (linked to the oxidation level) increases with temperature, which naturally results in higher oxygen content readings.

A visual inspection, as well as SEM observation, revealed the formation of substantial dot-like oxides on the surface polished with 50% to 80% of overlap in ambient air (a similar phenomenon was previously seen on IN718 by Bures and Zetek [32], but it is yet to be characterized). From 85% to 90%, the effect of these oxides can be considered to be minor due to the fact they mostly appear on the edges of the polished areas. For the samples polished in the argon chamber, a similar effect was not noticed. Therefore, further characterization was performed on two representative samples (1.o and 5.o).

**Figure 5:**
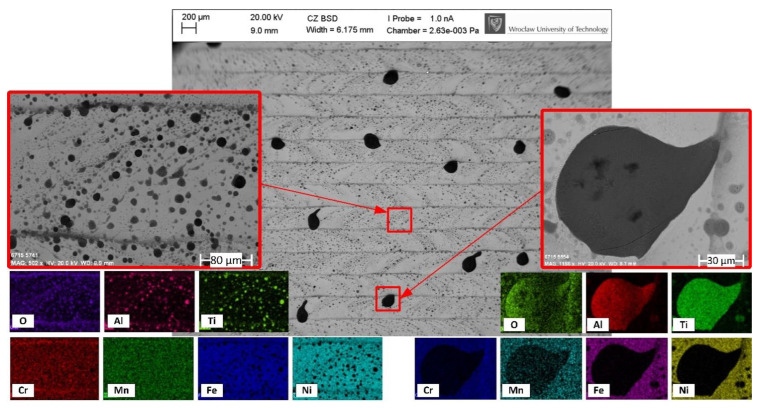
Back-scattered electron graph and EDS mapping showing the top-view and chemical elements of the sample (1.o) polished with 50% overlap in the air atmosphere.

The oxidation tendency of the elements included in IN718 is greatest for Al, Cr, Fe, Ti, and Ni, respectively, according to the Ellingham diagram. Therefore, these elements are most likely to form Al_2_O_3_, Cr_2_O_3_, FeO, TiO_2_, and NiO on the IN718 surface [49]. Further EDS investigations of 1.o sample (Figure 5) revealed that Al and Ti oxides can appear in the form of both single spots with a diameter more than 100 µm (Figure 5 right), and multiple small spots with a diameter of approximately 10 µm (Figure 5 left). After utilizing the high contrast in the photo, these oxides appear to be black, while the white-grey areas can be considered to be the least oxidize, since their chemical composition is close to the original IN718. As shown in the example in Figure 5 (right), microcracks occur on larger oxides, while, on smaller ones (Figure 5 left), this effect was not noticed. The formation of the of Al_2_O_3_ and TiO_2_ on IN718 in the melting regime was previously noticed by Gasper et al. in the context of the Laser Powder Bed [50], by Sanviemvongsak et al. in the context of Laser Beam Melting [39], and by Zhang et al. in the context of Direct Laser Deposition [49]. However, the authors, in their papers, utilized shielding gas and, therefore, these oxides appeared to be much smaller in size than the ones that were observed in this study, with similar cracking effects not being observed. A possible explanation for the formation of such large Ti and Al oxides could be related to the surface tension and temperature on the surface. When no shielding atmosphere is applied, the formation of these oxides is massively increased and they do not diffuse into bulk, because their melting point could not be reached (2072 °C for Al_2_O_3_ and 1843 °C for TiO_2_). Finally, these oxides can accumulate into larger structures due to the surface tension. 

**Figure 6:**
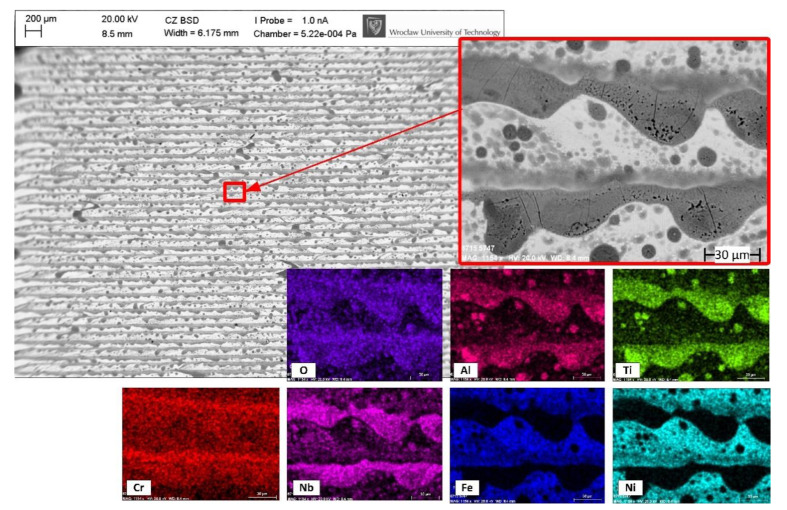
Back-scattered electron graph and EDS mapping showing the top-view and chemical elements of the sample (5.o) polished with 85% overlap in the air atmosphere.

For the 5.o trial (Figure 6), large Ti/Al oxides are no longer seen. However, the surface is clearly more oxidized, which generally confirms the results that are shown in Figure 4. Ti and Al oxides are still present, but they appear in the form of a longitudinal structure along the laser tracks and as randomly positioned small spots. The increasing overlap caused the increasing of energy density and local heat accumulation and, thus, most likely resulted in an increased maximum temperature reached on the surface [51,52]. This caused the melting point of Al_2_O_3_ and TiO_2_ to be reached, in turn allowing for oxides to diffuse and form different structures under the action of the subsequent laser scans. Moreover, the larger size oxides seen in 1.o are still present in the edges of the polished zone, which confirms the statement that they can only be diffused into a surface if higher overlaps are applied. In the case of higher overlap values, the formed Ti and Al oxides are significantly affected by micro-cracking, which is visible all along the dark-gray, longitudinal structures (Figure 6 right).

**Figure 7:**
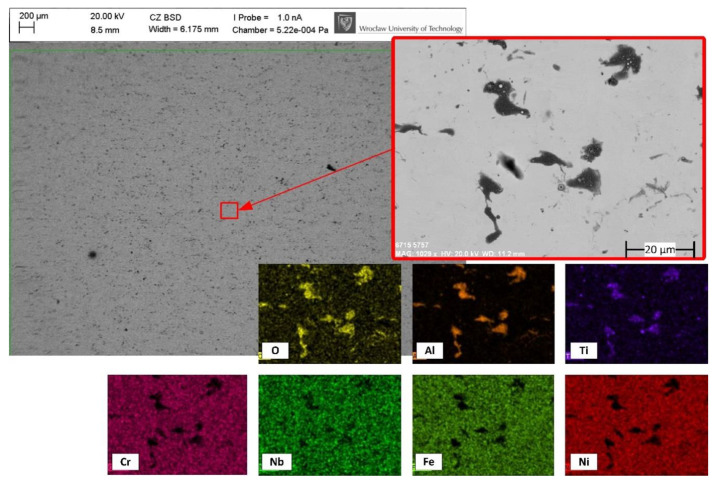
Back-scattered electron graph and EDS mapping showing the top-view and chemical elements of the sample (5.a) polished with 85% overlap in the argon chamber.

The values of the equilibrium partial pressures of the oxygen at the melting point for the aluminum and titanium is 10^−58^ atm and 10^−10^ atm, respectively, which means that the oxygen content inside the argon process chamber should be lower than 10^−52^ ppm (for Al) and 10^−4^ ppm (for Ti) in order to completely avoid the formation of Al_2_O_3_ and TiO_2_. When considering industry-grade inert gas, this rigor is clearly impossible to fulfil in non-lab conditions [49,50]. As expected, the 25 ppm oxygen content utilized in the process chamber was not low enough to prevent the aluminum and titanium from oxidation. Moreover, these oxides can rarely be spotted on samples processed in an argon atmosphere, which can be seen on the representative sample from trial 5.a (Figure 7). The microcracking effect was not noticed in the samples that were polished in the argon chamber.

The formation of the oxide layer might have significant influence on the mechanical as well as the tribological performance due to the current knowledge. Thus, from the point of view of full implementation of this approach to industry scale, it is crucial to investigate the performance of substrates polished in air atmosphere, in comparison with substrates polished in argon chamber.

The oxides that were obtained in this research, due to their morphology and distribution, will obviously have an impact on the fatigue-creep performance, but not so strong as oxides on grain boundaries that lead to the grain boundaries embrittlement. The influence of surface oxide on the mechanical properties of IN718 is quite complex and is still the subject of much research. IN718 is generally used in aircraft engines and turbine blades and it is commonly employed at operating temperatures below 650 °C [53]. Under these conditions, one of the most important properties is fatigue-creep performance. The nucleation of cavities is usually observed on grain boundaries and associated with the existence of second-phase particles. Grain-boundary sliding and dislocation pile-ups are two of the main mechanisms that control the nucleation of cavities [54]. Additively manufactured IN718 oxidized at 850 °C in air for 1000 h forms a 3 to 4 µm thick layer of oxides on the surface [39]. What is more important, intergranular oxidation appeared with depth from 6.5 to 8 µm below the surface. This oxide located in the grain boundary is mainly composed of titanium oxide near the oxide scale/alloy interface and alumina oxide, but deeper in the alloy. In general, the oxide-induced embrittlement increased at the grain boundaries, leads to the aggravation of creep damage, thus, fatigue-crack propagation accelerated. Moreover, the effect of creep and oxidation increased significantly with the increase of hold-time [55,56]. Hence, the effect of creep is complicated and it depends on different alloys, temperature, and environment, etc. [55].

The ceramic layer on the surface of Inconel 718 can have a very beneficial effect on hardness and wear resistance due to its properties. An example can be the nitrided layer on the IN718 surface [57,58]. Xue et al. showed that the nitrided layer achieves a hardness that is several times higher than that of Inconel (1800 HV0.01 and about 400 HV, respectively). Moreover, the results presented by Authors, prove that the nitrided layer strongly improves abrasive wear resistance [57]. Based on that, it is expected that the oxide layer formed in this study might have a beneficial effect on increased hardness and wear resistance, which is promising course for future studies.

### 3.2. Influence of Overlap

**Figure 8:**
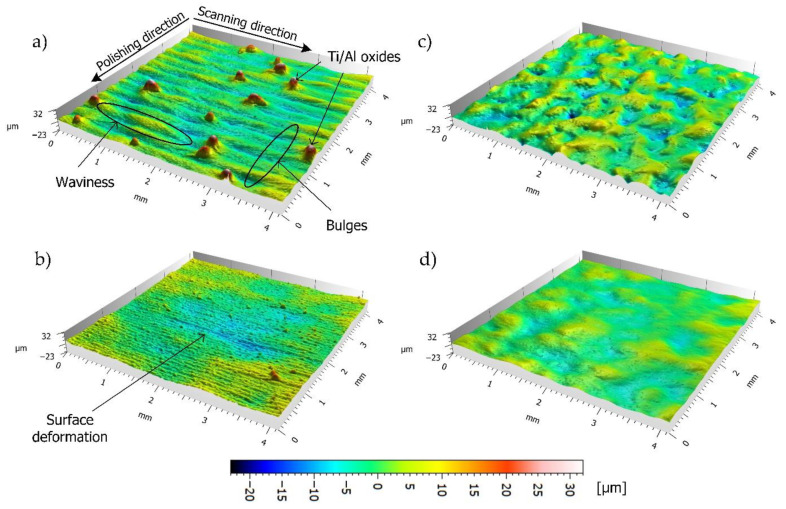
Laser Polished surface topographies recorded by white-light interferometry: (**a**) OL = 50% (air, 1.o), (**b**) OL = 85% (air, 5.o), (**c**) OL = 50% (argon, 1.a), and (**d**) OL = 85% (argon, 5.a).

**Figure 9:**
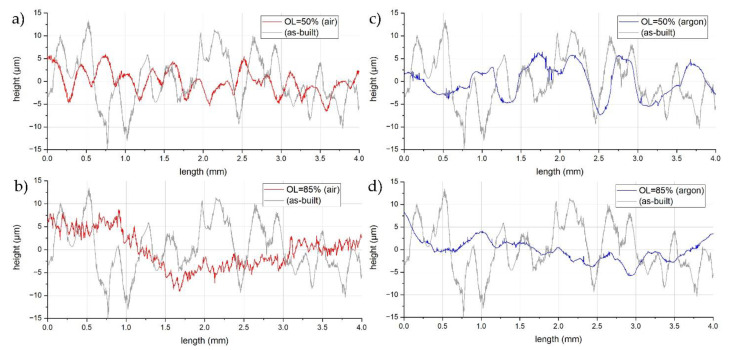
Single profiles perpendicular to the scanning direction, extracted from the topographies (Figure 8): (**a**) OL = 50% (air, 1.o), (**b**) OL = 85% (air, 5.o), (**c**) OL = 50% (argon, 1.a), and (**d**) OL = 85% (argon, 5.a).

When utilizing the zigzag strategy, overlap is a vital parameter that influences both the average roughness and periodic surface components [23]. Therefore, the experiment that was described in this chapter was conducted for the argon and oxygen atmospheres in accordance with Table 2. The laser power and scanning velocity was kept constant at 160 W and 150 mm/s, respectively. Figure 8 outlines the isometric views (Talymap) of four representative samples that were polished using 50% and 85% overlap in the air (Figure 8a,b) and argon (Figure 8c,d) atmospheres. However, Figure 9 shows representative profiles, perpendicular to the scanning direction (which was taken from the topographies that are presented in Figure 8), which were compared with the profile from the as-built sample (gray curve). LP in the air atmosphere results in a higher heat input due to the fact that the convection losses are generally less than the ones that were processed in the argon chamber. Thus, the process parameters were sufficient enough to perform full re-melting of the asperities and, as a result of material redistribution, a new structure was formed. Newly formed surface features can be identified as a product of the SOM mechanism. Figure 8a and Figure 9a outline the formation of low frequency surface features along the laser scanning direction under the action of thermo-capillary oscillations that are caused by the surface tension gradient occurring during the laser scanning [10]. On the other hand, during laser re-melting, material tends to expand as a result of plastic deformation, and then shrinks during solidification. This causes the formation of bulges, which, in combination with the zigzag strategy, results in the shaping of the periodic structure perpendicular to the scanning direction. However, when overlap is high enough, this effect is no longer dominant, which proves the findings presented by Nüsser et al., as shown in Figure 8b and Figure 9b [14]. When higher overlaps are applied, energy density is increased exponentially, which results in higher local heat accumulation on the surface. Therefore, the surface is more likely to deform, as shown in Figure 8b, where the amplitude in the valley reaches 12.6 µm from the reference line. In the case of the samples that were polished in the argon chamber (Figure 8c,d), the formation of the surface lay pattern related to scanning direction is no longer seen. Instead, surface asperities are only partially redistributed, and surface behavior can be identified with the SSM mechanism, although the transition between these mechanisms is not clearly defined in the literature.

**Figure 10:**
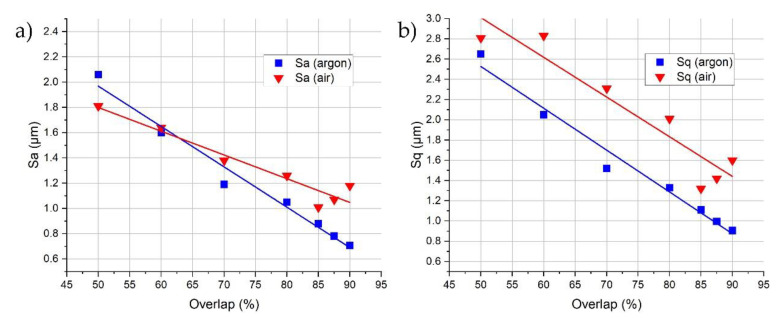
Evaluation of Sa (**a**) and Sq (**b**) surface roughness parameters for the trials Laser Polished in accordance with Table 2.

Roughness analysis (Figure 10) reveals that an almost equal mean surface roughness (Sa) is achieved for OL = 50% for both the argon and air atmospheres (2.06 and 1.81 µm, respectively). For the argon trial (1.a), the increasing overlap results in an almost linearly decreased Sa. Moreover, the best results can be achieved for higher overlaps, which generally proves the findings that were presented by Rosa et al. [24], Bhaduri et al. [21], and Hafiz et al. [23]. However, in the case of air, the lowest roughness is achieved for 85% overlap, which is contrary to the argon experiment, in which the roughness increases above this value. This may be a result of the surface overheating and melt-pool instability.

Furthermore, Sa is not a suitable roughness parameter for describing surfaces that are characterized by single peaks/valleys that are high and narrow enough to not be cut-off by a waviness filter. Figure 10a shows that the occurrence of Ti/Al oxides (the height of which can reach up to 32 µm) did not increase the Sa values significantly (Sa (air)). However, when the standard deviation of height (Sq) is used (Figure 10b), the samples that are polished in the argon chamber show a trend similar to the Sa, while the samples polished in the air atmosphere are significantly different for the trials where Ti/Al oxides occur (50%–80% overlaps).

**Figure 11:**
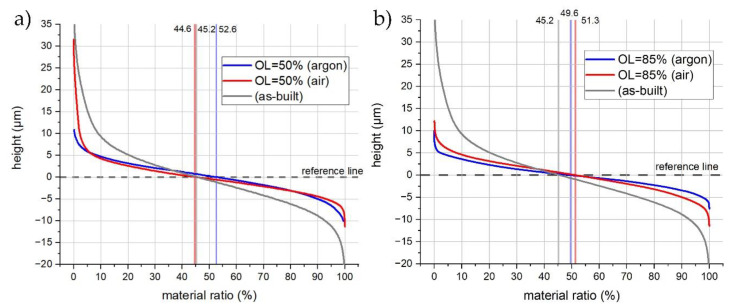
Abbott-Firestone curves for the samples polished in argon and air with OL = 50% (**a**) and OL = 85% (**b**).

The material’s relocation mechanism is linked with the laser thermal energy process (LP) and it might lead to the non-uniformity of the distribution of amplitudes (peaks and valleys). This is the result of the forming of material- and process-induced surface structures during melt-pool re-solidification [14,23]. Therefore, the Abbott–Firestone curve can be used to obtain information regarding the percentage distribution of the asperities over the analyzed surface. For the as-built sample (Figure 11a,b, gray), 45.2% of the material is localized above the reference line, and 54.8% of the material is localized below the reference line. The curve is characterized by similar slopes in the upswing and downswing regions. After applying LP with OL = 50% in the air atmosphere (Figure 11a, red), the ratio changed insignificantly to 44.6% and 55.4% for the above and below reference lines, respectively. However, the slope in the upswing is steeper due to the occurrence of Ti/Al oxides (very few objects with a high amplitude), while, in the downswing, the slope is decreased due to the formation of bulges. Conducting the process in the argon chamber (Figure 10a, blue) resulted in a reduced heat input being applied to the surface, therefore causing the smoothing of the as-built surface. The slope of the up- and downswing became symmetrical, and the amplitude distribution ratio reached 52.6% (above) and 47.4% (below). When the overlap was increased to 85%, a uniform surface distribution was achieved for LP in both the air (51.3% and 48.7%) and argon (49.6% and 50.4%) atmospheres, and the slope of the middle section was decreased. However, the slope of the downswing, representing LP in the air atmosphere (Figure 10b, red), was not reduced when compared to OL = 50% (Figure 11a, red). This might be due to the thermal deformation of the sample as a result of the high heat input. It can be concluded that for high overlap (85%), in both the argon and air approaches, a similar amount of material occurs above and below the reference line and, therefore, the distribution of amplitudes is generally more uniform. Moreover, the surface quality was improved in all the LP trials, which is represented by the lowered slope of the middle section curve.

**Figure 12:**
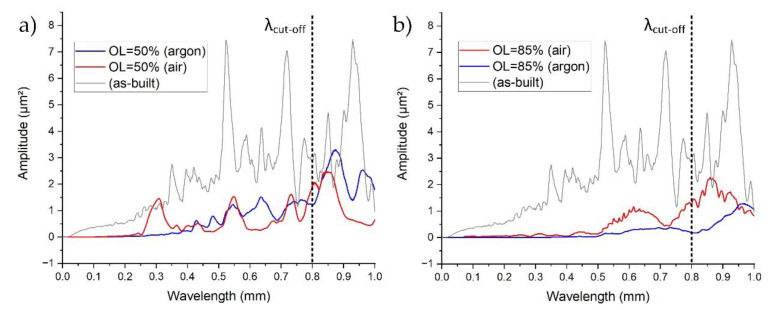
Power Spectral Density functions for the samples polished in argon and air with OL = 50% (**a**) and OL = 85% (**b**).

When the zigzag strategy is applied, periodic surface components will change with the utilized overlap [23]. In this case, the PSD function was used in order to show the distribution of power of all the periodic components on the surface created after the LP in the direction perpendicular to the scanning direction. Additionally, the horizontal axis was inverted from the frequency to the wavelength domain (λ = 1/f) for a better understanding of the occurring components. Figure 12a shows that, for the sample polished in the argon chamber, higher frequencies (shorter wavelengths) are significantly reduced when compared to the as-built surface. However, a high gain is still observed above the cut-off value (waviness). The same process that was conducted in the air atmosphere resulted in the amplification of the wavelength by approximately 0.3 µm (equal to the hatching pitch), which is the direct result of the formation of periodic bulges (see Figure 8a and Figure 9a). Increasing the overlap to 85% resulted in a significant reduction in higher frequencies for the LP that was conducted in the argon chamber (Figure 12b, blue). For the LP conducted in air (Figure 12b, red), the peak at 0.3 µm was reduced due to the fact that the periodic bulge structures are no longer dominant. However, lower frequencies were not reduced, but instead amplified. This is due to surface overheating, which results in surface deformation and, in turn, increased waviness.

## 4. Discussion

**Figure 13:**
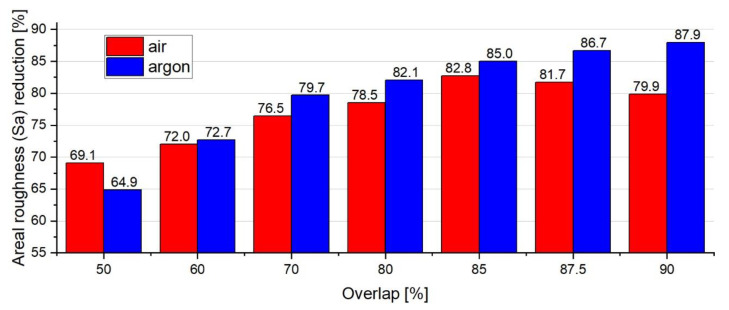
Comparison of the average areal roughness (Sa) reduction after applying LP under the effect of the air and argon atmospheres.

The issues that were covered in this paper were limited to the investigation of the influence of overlap on surface morphology and topography. Overlap is considered to be a crucial parameter in the case of the zigzag strategy. Further analysis consisting of the influence of process parameters on surface quality could be carried out using multivariate analysis (Design of Experiment). Finally, Table 3 shows a summary of observations of the obtained results with regards to the overlap and the impact of gas. Moreover, Figure 13 shows the average reduction in roughness (Sa) (as a function of overlap) that is possible to achieve when conducting LP in air and argon atmospheres. 

## 5. Conclusions

It was proven that, in the context of roughness reduction, LP can be a sufficient polishing method of IN718 in both an air and argon environment. However, not using the chamber saves costs and increases the versatility of the process, but it is associated with problems that concern the formation of the oxide layer. The formation of an oxidation layer imposes a narrow parameter window and it could be more unpredictable when compared to the quiet and more stable argon chamber variant. Moreover, the process conducted in air is much more vulnerable to contaminants in the air, which is vital when considering the repeatability of the process and the chemical composition of the formed oxidation layer. Reducing the surface roughness of IN718, by means of LP, without a shielding atmosphere is possible, but it has many issues and limitations that need to be taken into consideration. In this paper, the major issues that were related to the formation of the oxidation layer were identified and described in the context of Laser Polishing. Moreover, the analysis of the IN718 oxidation layer performed in this paper can be valuable in the context of other laser technologies that are based on similar process mechanisms, e.g., laser conduction welding or laser surface structuring.

In this paper, the novel approach of Laser Polishing of IN718 in air atmosphere was presented. The scope of research was limited to the characterization of formed oxide layer and an evaluation of the roughness parameters, in comparison with well-established LP in the argon chamber. The next step of this approach, leading to a full validation and industrialization of this technology, would be an investigation of the oxidation layer influence on the mechanical and tribological performance, in comparison with well-established LP in argon atmosphere.

## Figures and Tables

**Table 1:** SLM process parameters and the chemical composition of the IN718 powder.

SLM Manufacturing Parameters						
**Beam Focus**	**Scan Hatching**		**Scanning Speed**	**Power**	**Layer (Thickness)**	
100 µm	80 µm		1300 mm/s	300 W	30 µm	
Strategy						
**Strategy**	**Scan Rotation Angle**		**Scan Vector Rotation Limitation Window**			
Stripes 7 mm	45°		From 45° and 135° (in relation to the gas flow vector)			
IN718 powder chemical composition in wt%						
**Ni**	**Cr**	**Nb**	**Mo**	**Ti**	**Co**	**Al**
50–55	17–21	4.75–5.50	2.80–3.30	0.65–1.15	1.00	0.20–0.80
**Si, Mn**	**Cu**	**C**	**B**	**P, S**	**Fe**	–
0.35 each	0.3	0.08	0.006	0.015 each	balance	–

**Table 2:** Parameter plan for the overlap influence experiment.

Test No.	Process Gas	Overlap %	Energy Density J/mm^2^	Laser Power W	Scanning Speed mm/s
1.a/1.o	Argon (a)/Oxygen (o)	50	7.11	160	150
2.a/2.o		60	11.11		
3.a/3.o		70	19.75		
4.a/4.o		80	44.44		
5.a/5.o		85	79.01		
6.a/6.o		87.5	113.78		
7.a/7.o		90	177.78		

**Table 3:** Summary of the influence of overlap on surface features and process efficiency after applying Laser Polishing in the air and argon atmospheres.

Criteria	Gas	Overlap [%]						
		50	60	70	80	85	87.5	90
Visual inspection	Air	No gloss effect. Visible individual laser tracks (bulges) and Ti/Al oxides.				No gloss effect. Individual laser tracks not visible.		
	Argon	The higher the overlap, the higher the gloss effect. Individual laser tracks not visible.						
Oxidation layer	Air	Approximately 100 large Ti/Al oxides per 100 mm^2^ area. Microcracks.				Ti/Al oxides mainly on the edges of the LP area. Microcracks.		
	Argon	Low slope linear oxidation. Occurrence of minor Ti/Al oxides. No microcracking.						
Power Spectral Density	Air	Peaks at wavelengths equal to the hatching pitch (bulges)				Peaks at longer wavelengths (surf. deform.)		
	Argon	Shorter wavelengths (roughness) decreased. Peaks at the wavelength above cut-off (surf. waviness)						
Abbott-Firestone	Air	Steep slope at upswing (Ti/Al oxides). Low mid. section slope				Steep slope at downswing (surf.deform.). Low mid slope		
	Argon	Uniform amplitude distribution (similar upswing and downswing). Low mid. section slope.						
Process efficiency [mm^2^/s]	Air	45	36	27	18	13.5	11.25	9
	Argon							

## Data Availability

The data presented in this study are available on request from the corresponding author.

## References

[1] Abdulhameed O., Al-Ahmari A., Ameen W., Mian S.H. (2019). Additive Manufacturing: Challenges, trends, and applications. Adv. Mech. Eng..

[2] Kruth J.P., Froyen L., van Vaerenbergh J., Mercelis P., Rombouts M., Lauwers B. (2004). Selective laser melting of iron-based powder. J. Mater. Process. Technol..

[3] Kurzynowski T., Stopyra W., Gruber K., Ziółkowski G., Kuźnicka B., Chlebus E. (2019). Effect of scanning and support strategies on relative density of SLM-ed H13 steel in relation to specimen size. Materials.

[4] Brynk T., Pakiela Z., Ludwichowska K., Romelczyk B., Molak R.M., Plocinska M., Kurzac J., Kurzynowski T., Chlebus E. (2017). Fatigue crack growth rate and tensile strength of Re modified Inconel 718 produced by means of selective laser melting. Mater. Sci. Eng. A.

[5] Udroiu R., Braga I.C., Nedelcu A. (2019). Evaluating the quality surface performance of Additive Manufacturing systems: Methodology and a material jetting case study. Materials.

[6] Kim U.S., Park J.W. (2019). High-quality surface finishing of industrial three-dimensional metal Additive Manufacturing using electrochemical polishing. Int. J. Precis. Eng. Manuf. Green Tech..

[7] Wang J., Zhu J., Liew P.J. (2019). Material removal in ultrasonic abrasive polishing of additive manufactured components. Appl. Sci..

[8] Shao T.M., Hua M., Tam H.Y., Cheung E.H. (2005). An approach to modelling of Laser Polishing of metals. Surf. Coat. Technol..

[9] Mohajerani S., Bordatchev E.V., Tutunea-Fatan O.R. (2018). Recent developments in modeling of Laser Polishing of metallic materials. Lasers Manuf. Mater. Process..

[10] Ramos J.A., Bourell D.L., Beaman J.J. (2002). Surface over-melt during Laser Polishing of indirect-SLS metal parts. MRS Proc..

[11] Poprawe R. (2011). Tailored Light 2.

[12] Krishnan A., Fang F. (2019). Review on mechanism and process of surface polishing using lasers. Front. Mech. Eng..

[13] Marimuthu S., Triantaphyllou A., Antar M., Wimpenny D., Morton H., Beard M. (2015). Laser Polishing of selective laser melted components. Int. J. Mach. Tools Manuf..

[14] Nüsser C., Kumstel J., Kiedrowski T., Diatlov A., Willenborg E. (2015). Process- and material-induced surface structures during Laser Polishing. Adv. Eng. Mater..

[15] Vadali M., Ma C., Li X., Pfefferkorn F.E., Duffie N.A. (2018). Intelligent scan trajectories for pulsed Laser Polishing. Int. J. Mechatron. Manuf. Syst..

[16] Flemmer J., Ross I., Willenborg E., Fröba H. (2015). Machine tool and CAM-NC data chain for Laser Polishing complex shaped parts. Adv. Eng. Mater..

[17] Zhou Y., Zhao Z., Zhang W., Xiao H., Xu X. (2019). Experiment study of rapid Laser Polishing of freeform steel surface by dual-beam. Coatings.

[18] Caggiano A., Teti R., Alfieri V., Caiazzo F. (2021). Automated Laser Polishing for surface finish enhancement of additive manufactured components for the automotive industry. Prod. Eng. Res. Devel..

[19] Alfieri V., Argenio P., Caiazzo F., Vincenzo S. (2016). Reduction of surface roughness by means of laser processing over Additive Manufacturing metal parts. Materials.

[20] Fan W., Yang Y., Lou R., Chen X., Bai J., Cao W., Cheng G., Si J. (2019). Influence of energy fluence and overlapping rate of femtosecond laser on surface roughness of Ti-6Al-4V. Opt. Eng..

[21] Bhaduri D., Penchev P., Batal A., Dimov S., Soo S.L., Sten S., Harrysson U., Zhang Z., Dong H. (2017). Laser Polishing of 3D printed mesoscale components. Appl. Surf. Sci..

[22] Rosa B., Hascoet J.Y., Mognol P. (2014). Modeling and optimization of Laser Polishing process. AMM.

[23] Hafiz A.M.K., Bordatchev E.V., Tutunea-Fatan R.O. (2012). Influence of overlap between the laser beam tracks on surface quality in Laser Polishing of AISI H13 tool steel. J. Manuf. Process..

[24] Rosa B., Mognol P., Hascoët J. (2016). Modelling and optimization of Laser Polishing of additive laser manufacturing surfaces. Rapid Prototyp. J..

[25] Yung K.C., Wang W.J., Xiao T.Y., Choy H.S., Mo X.Y., Zhang S.S., Cai Z.X. (2018). Laser Polishing of additive manufactured CoCr components for controlling their wettability characteristics. Surf. Coat. Technol..

[26] Bustillo A., Ukar E., Rodriguez J.J., Lamikiz A. (2011). Modelling of process parameters in Laser Polishing of steel components using ensembles of regression trees. Int. J. Comp. Integr. Manuf..

[27] Pilipović A., Brajlih T., Drstvenšek I. (2018). Influence of processing parameters on tensile properties of SLS polymer product. Polymers.

[28] Temmler A., Liu D., Preußner J., Oeser S., Luo J., Poprawe R., Schleifenbaum J.H. (2020). Influence of Laser Polishing on surface roughness and microstructural properties of the remelted surface boundary layer of tool steel H11. Mater. Design.

[29] Temmler A., Ross I., Luo J., Jacobs G., Schleifenbaum J.H. (2020). Influence of global and local process gas shielding on surface topography in laser micro polishing (LμP) of stainless steel 410. Surf. Coat. Technol..

[30] Perry T.L., Werschmoeller D., Li X., Pfefferkorn F.E., Duffie N.A. (2009). Pulsed Laser Polishing of micro-milled Ti6Al4V samples. J. Manuf. Process..

[31] dos Santos Solheid J., Jürgen Seifert H., Pfleging W. (2018). Laser surface modification and polishing of additive manufactured metallic parts. Proc. CIRP.

[32] Bures M., Zetek M. (2020). Application of laser surface polishing on additvie manufactured parts of inconel 718 nickel-based superalloy. MM SJ.

[33] Perry T.L., Werschmoeller D., Duffie N.A., Xiaochun L., Pfefferkorn F.E. (2009). Examination of selective pulsed laser micropolishing on microfabricated nickel samples using spatial frequency analysis. J. Manuf. Sci. Eng..

[34] Willenborg E. (2006). Polieren von Werkzeugstählen mit Laserstrahlung.

[35] Temmler A., Liu D., Luo J., Poprawe R. (2020). Influence of pulse duration and pulse frequency on micro-roughness for laser micro polishing (LµP) of stainless steel AISI 410. Appl. Surf. Sci..

[36] Ross I., Temmler A., Willenborg E., Poprawe R., Teller M. (2015). Investigation of the influence of laser surface modifications on the adhesive wear behavior in dry cold extrusion of aluminum. Lasers Manuf. Conf..

[37] Paulonis D.F., Schirra J.J. (2001). Alloy 718 at Pratt & Whitney–Historical perspective and future challenges. Superalloys.

[38] Zhihao F., Libin L., Longfei C., Yingchun G. (2018). Laser Polishing of additive manufactured superalloy. CIRP Conf. Surf. Integr..

[39] Sanviemvongsak T., Monceau D., Macquaire B. (2018). High temperature oxidation of IN 718 manufactured by laser beam melting and electron beam melting: Effect of surface topography. Corros. Sci..

[40] Kumstel J., Kirsch B. (2013). Polishing titanium- and nickel-based alloys using Cw-laser radiation. Phys. Proc..

[41] Hu G., Song Y., Guan Y. (2019). Tailoring metallic surface properties induced by laser surface processing for industrial applications. Nanotechnol. Precis. Eng..

[42] Dadbakhsh S., Hao L., Kong C.Y. (2010). Surface finish improvement of LMD samples using Laser Polishing. Virtual Phys. Prototyp..

[43] Temmler A., Schmickler T., Willenborg E. Surface structuring by laser remelting of Inconel 718. Proceedings of the Lasers in Manufacturing Conference.

[44] Zhang D., Yu J., Li H., Zhou X., Song C., Zhang C., Shen S., Liu L., Dai C. (2020). Investigation of Laser Polishing of four selective laser melting alloy samples. Appl. Sci..

[45] Fraunhofer Institute for Laser Technology ILT Laser Polishing of SLM Components out of Inconel 718. www.ilt.fraunhofer.de.

[46] Greene G.A., Finfrock C.C. (2001). Oxidation of inconel 718 in air at high temperatures. Oxid. Metals.

[47] Yu H., Hayashi S., Kakehi K., Kuo Y.-L. (2019). Study of formed oxides in IN718 alloy during the fabrication by selective laser melting and electron beam melting. Metals.

[48] Temmler A., Comiotto M., Ross I., Kuepper M., Liu D.M., Poprawe R. (2019). Surface structuring by laser remelting of 1.2379 (D2) for cold forging tools in automotive applications. J. Laser Appl..

[49] Zhang Y.N., Cao X., Wanjara P., Medraj M. (2013). Oxide films in laser additive manufactured Inconel 718. Acta Mater..

[50] Gasper A., Szost B., Wang X., Johns D., Sharma S., Clare A.T., Ashcroft I.A. (2018). Spatter and oxide formation in laser powder bed fusion of Inconel 718. Addit. Manuf..

[51] Guan Y.C., Zhou W., Li Z.L., Zheng H.Y. (2013). Influence of overlapping tracks on microstructure evolution and corrosion behavior in laser-melt magnesium alloy. Mater. Design.

[52] Martan J., Cibulka O., Semmar N. (2006). Nanosecond pulse laser melting investigation by IR radiometry and reflection-based methods. Appl. Surf. Sci..

[53] Schafrik R.E., Ward D.D., Groh J.R. (2001). Application of alloy 718 in GE aircraft engines: Past, present and next five years. Superalloys 718, 625, 706 and Various Derivatives (2001).

[54] Kassner M.E. (2009). Fundamentals of Creep in Metals and Alloys.

[55] Wang M., Du J., Deng Q. (2020). The Mechanism of creep during crack propagation of a superalloy under fatigue-creep-environment interactions. Materials.

[56] Li X., Dong J.X., Zhang L.N., Guo W.M. (2001). Effects of fatigue dwell time and solid solution on crack growth rate of PM Rene’ 95 superalloy at high temperature. Acta Metall. Sin..

[57] Xue L., Wang J., Li L., Chen G., Sun L., Yu S. (2020). Enhancement of wear and erosion-corrosion resistance of Inconel 718 alloy by liquid nitriding. Mater. Res. Express.

[58] Maniee A., Mahboubi F., Soleimani R. (2020). Improved hardness, wear and corrosion resistance of inconel 718treated by hot wall plasma nitriding. Met. Mater. Int..

